# Pyruvate Dehydrogenase Kinases in the Nervous System: Their Principal Functions in Neuronal-glial Metabolic Interaction and Neuro-metabolic Disorders

**DOI:** 10.2174/157015912804143586

**Published:** 2012-12

**Authors:** Mithilesh Kumar Jha, Sangmin Jeon, Kyoungho Suk

**Affiliations:** Department of Pharmacology, Brain Science & Engineering Institute, Kyungpook National University School of Medicine, Daegu, Korea

**Keywords:** Aerobic glycolysis, neuro-metabolic disorders, neuronal-glial interaction, oxidative phosphorylation, pyruvate dehydrogenase complex, pyruvate dehydrogenase kinase.

## Abstract

Metabolism is involved directly or indirectly in all processes conducted in living cells. The brain, popularly viewed as a neuronal–glial complex, gets most of its energy from the oxygen-dependent metabolism of glucose, and the mitochondrial pyruvate dehydrogenase complex (PDC) plays a key regulatory role during the oxidation of glucose. Pyruvate dehydrogenase kinase (also called PDC kinase or PDK) is a kinase that regulates glucose metabolism by switching off PDC. Four isoforms of PDKs with tissue specific activities have been identified. The metabolisms of neurons and glial cells, especially, those of astroglial cells, are interrelated, and these cells function in an integrated fashion. The energetic coupling between neuronal and astroglial cells is essential to meet the energy requirements of the brain in an efficient way. Accumulating evidence suggests that alterations in the PDKs and/or neuron-astroglia metabolic interactions are associated with the development of several neurological disorders. Here, the authors review the results of recent research efforts that have shed light on the functions of PDKs in the nervous system, particularly on neuron-glia metabolic interactions and neuro-metabolic disorders.

## 1 INTRODUCTION

Pyruvate dehydrogenase complex (PDC) having a predominant role in the regulation of mammalian metabolism represents the point-of-no-return regarding the utilization of carbohydrate, plays a leading role in maintaining glucose homoeostasis and is immensely involved in the metabolic pathway required for energy production and movement [1-3]. PDC is entirely nuclear encoded and consists of multiple copies of three structurally distinct, but functionally interdependent, enzymes (E_1_ through E_3_) [4]. Mammalian PDC is a huge complex with several components: pyruvate dehydrogenase (E1), dihidrolipoyl transactylase (E2), E3 binding domain (E3BP) and dihydrolipoyl dehydrogenase (E3) [4-8]. Pyruvate dehydrogenase (E1) is the first component enzyme of PDC.

Pyruvate is the end product of glycolysis in the cytosol. Glycolysis, a series of enzymatically catalyzed reactions occurring within cells, by which glucose and other sugars are broken down to yield lactic acid or pyruvic acid, releasing energy in the form of ATP, is well differentiated into two types on the basis of oxygen consumption during the reaction. The conversion of glucose to lactate in the presence of oxygen has been termed aerobic glycolysis, whereas the conversion of glucose to lactate in the absence of oxygen has been termed anaerobic glycolysis. Recently, it was hypothesized that, in the brain, both aerobic and anaerobic glycolysis terminate with the formation of lactate from pyruvate by lactate dehydrogenase (LDH). If this hypothesis is correct, the lactate must be the mitochondrial substrate for oxidative energy metabolism *via *its oxidation to pyruvate, possibly *via *mitochondrial LDH. A new finding supports the hypothesis that lactate, at least in an *in vitro* setting, is indeed the principal end product of neuronal aerobic glycolysis [9].

Under aerobic conditions, pyruvate enters the mitochondria, where it is transformed to acetyl-coenzyme A (acetyl-CoA), producing dihydronicotinamide adenine dinucleotide (NADH) and carbon dioxide. Acetyl-CoA subsequently enters the Krebs (citric acid) cycle, and thus, provides energy (adenosine triphosphate or ATP) to the cell [10]. This reaction is catalyzed by the enzyme-coenzyme complex PDC. PDC is located in the mitochondrial matrix space, and is responsible for irreversibly converting pyruvate into acetyl CoA, the primary fuel of the citric acid cycle (CAC). Reactions of the CAC and fatty acid oxidation are performed in the mitochondrial matrix. The mitochondrial PDC reaction connects glycolysis to oxidative metabolism to provide oxidative fuel for the generation of ATP. The PDC reaction also provides acetyl-CoA for fatty acids synthesis in fat synthesizing tissues when carbohydrate intake is excessive. PDC activity is under the control of pyruvate dehydrogenase kinases (also pyruvate dehydrogenase complex kinases, PDC kinases, or PDKs) 1 to 4, which phosphorylate the E1 subunit of PDC and suppress the catalysis of pyruvate to acetyl-CoA [11]. The activity of PDK is regulated by the concentrations of the metabolic products of pyruvate (NADH and acetyl-CoA). The PDH/PDK system acts as a key regulator of mitochondrial activity and plays an important role in the switching of the metabolism from oxidative phosphorylation to aerobic glycolysis that accompanies malignant transformation.

**Fig. (1):**
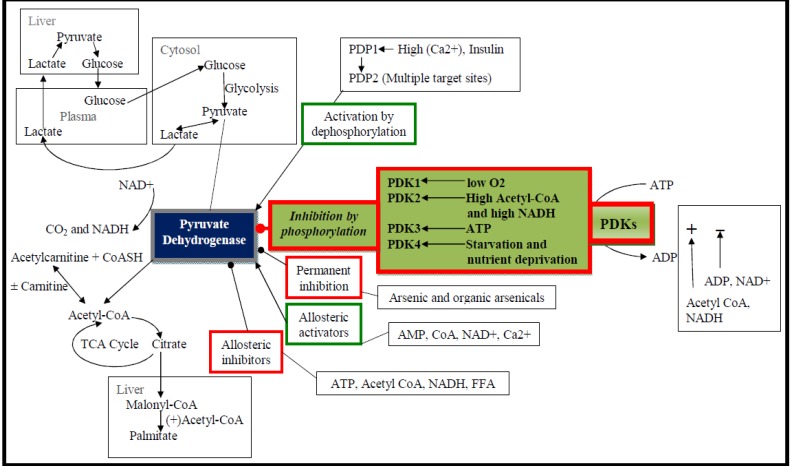
**The foremost tasks of PDK and PDP in the regulation of PDC and related metabolic processes**. PDC is a large multi-enzyme complex located within the matrix compartment of mitochondria, and links glycolysis to the tricarboxylic acid cycle by catalyzing the irreversible oxidative decarboxylation of pyruvate, which leads to the generation of CO2, NADH, and acetyl-CoA. PDC activity is regulated by reversible phosphorylation. PDK inactivates PDC by phosphorylation. Conversely, PDP activates PDC by dephosphorylation. The regulation of PDC is linked to the metabolisms of glucose and fatty acid. PDC, pyruvate dehydrogenase complex; PDK, pyruvate dehydrogenase kinase; NADH, nicotinamide adenine dinucleotide hydride; FFA, free fatty acids; PDP, pyruvate dehydrogenase phosphatase; PEPCK; phosphoenolpyruvate carboxykinase; TCA, tricarboxylic acid cycle.

PDK isozymes together with the related branched chain dehydrogenase kinase comprise a novel family of serine kinases unrelated to cytoplasmic Ser/Thr/Tyr kinases [12-17]. PDKs are involved in the regulation of glucose oxidation, which could ultimately affect whole body glucose metabolism [18-20]. They consist of two subunits, that is, an α subunit with kinase activity, and a β, which is a regulatory subunit. PDK is a kinase enzyme that acts to inactivate PDC by phosphorylating it using ATP. PDK is involved in the regulation of the PDC activity (Fig. **1**), which catalyzes the oxidative decarboxylation of pyruvate to acetyl CoA [10]. Although PDC is regulated by several mechanisms, including allosteric inhibition by acetyl CoA and NADH, covalent modification of PDC is extremely important for the long-term regulation of metabolic processes. PDKs phosphorylate PDC, whereas pyruvate dehydrogenase phosphatases (PDPs) catalyze the reverse reaction. PDC is dephosphorylated and active in the well-fed state to facilitate the oxidation of carbohydrates, but PDC is phosphorylated and inactive in the starved state to conserve substrates required for gluconeogenesis [21]. When carbohydrate stores are reduced in mammals, PDC activity is down-regulated to limit the consumption of glucose *via *oxidative phosphorylation in tissues that can use fatty acids or ketone bodies, such as, heart and skeletal muscle. The important exception is neuronal tissue, which processes glucose almost exclusively for ATP production.

Hitherto, four tissue-specific isoforms (PDK 1–4) of PDK have been identified in the mammalian mitochondria [22]. PDK1 has been found in the heart [23, 24], pancreatic islets [25], and skeletal muscles [26]. PDK2 is ubiquitously expressed [27], whereas PDK3 has only been detected in the testes, kidneys, and brain [27], and PDK4 in the heart, skeletal muscles, liver, kidneys, brain, and pancreatic islets [23, 25, 26, 28]. PDKs phosphorylate three serine sites on PDC E1α, and whereas PDKs can phosphorylate sites 1 and 2, PDK1 uniquely phosphorylates site 3 [23, 25-27, 29, 30]. In rodents, there are two isoenzymic forms of PDK, which share up to 70% amino acid identity [31], and are designated PDK1 and PDK2. PDK4 was recently shown to have higher kinase activity that the other three PDK isoforms, regardless of the presence of L2 or the E2p/E3BP core [32]. Both isoenzymes, obtained as recombinant proteins, were found to be able of catalyze the phosphorylation and inactivation of PDC [31, 33]. Each PDK isoform has different specific activities and different sensitivities to pyruvate and ADP, which allows for individual responses to changing metabolic demands by targeting specific isoforms for upregulation [19, 34]. The existence of multiple isoenzymes of PDK in mammalian tissues suggests that their functionalities differ in particular tissues. Furthermore, the fraction of active PDC is reduced by the activities of dedicated PDK isoforms [35].

According to a recent report on protein expression in rat brain, PDK isoenzyme mRNAs are highly expressed in the cerebral cortex, hippocampus, amygdala, thalamus and other brain regions that could play important roles in regulating PDHE1α1 phosphorylation/dephosphorylation cycles and its activity in these regions under physiological conditions. In addition, PDK2 is the most abundant isoenzyme in the rat brain under physiological conditions, with PDK4 rarely expressed. The factors accounting for the regional heterogeneity of the isoforms and balance between phosphorylation and phosphatase activities remain unknown [36].

## 2 PDKs GOVERN THE ACTIVITY OF PDC

**Fig. (2):**
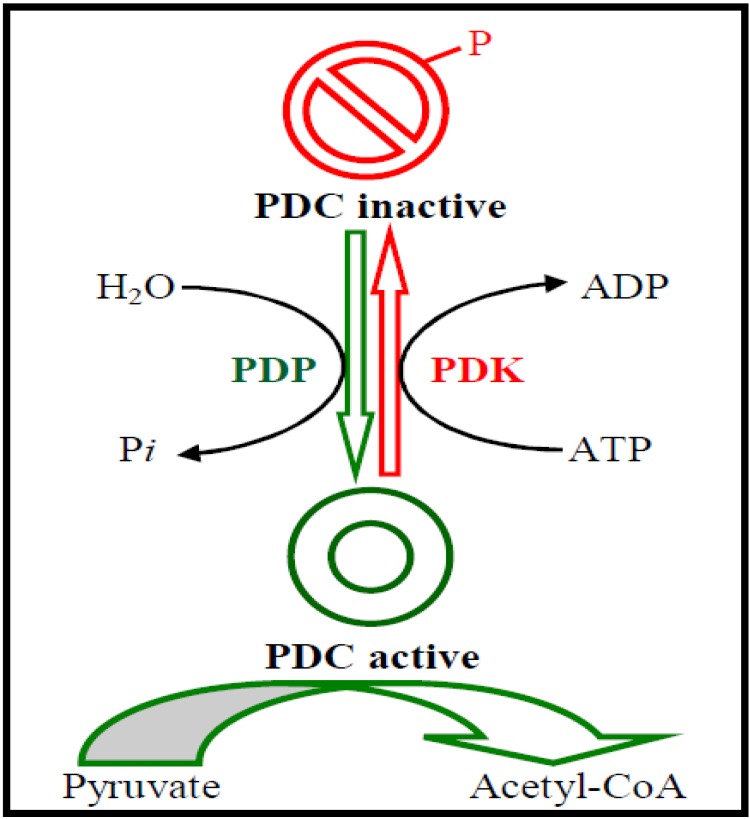
**Regulation of PDC by phosphorylation-dephosphorylation**. PDC catalyzes the oxidative decarboxylation of pyruvate, to form acetyl-CoA. PDKs catalyze the phosphorylations of serine residues of E1 of PDC, inhibiting the complex, whereas PDPs reverse this inhibition *via* dephosphorylation. PDC, pyruvate dehydrogenase complex; PDK, pyruvate dehydrogenase kinase; PDP, pyruvate dehydrogenase phosphatase; ADP, adenosine diphosphate; ATP, adenosine triphosphate.

PDK participates in the regulation of PDC in which PDH is the first component. PDK can phosphorylate a serine residue on PDH at three possible sites. Phosphorylation at site 1 nearly completely deactivates the enzyme while phosphorylation at sites 2 and 3 makes only a small contribution to the complex inactivation. Therefore, phosphorylation at site 1 is responsible for PDH deactivation. PDK inhibition by pyruvate facilitates PDH activation, favoring glucose oxidation and malonyl-CoA formation: the latter suppresses long-chain fatty acid (LCFA) oxidation. PDK activation by high mitochondrial acetyl-CoA/CoA and NADH/NAD(+) concentration ratios that reflect high rates of LCFA oxidation causes blockade of glucose oxidation [18, 37-39]. On the other hand, PDP removes the phosphate groups on PDH and reactivates the enzyme (Fig. **2**). To satisfy the discrete tissue-specific roles that PDC must fulfill to manage fuel consumption and storage, a set of dedicated regulatory enzymes provide highly adaptable control of the fraction of active PDC (PDCa) [11, 20, 40-48]. Four PDK isozymes [12, 15, 44, 47] govern the activity of PDC [35], and short-term and long-term mechanisms act to alter the activities and levels of PDK to manage the amount required for the storage of fuels [11, 20, 40-47]. It is reported that PDK is the effector that ultimately conveys the inhibitory signal from phosphorylated c-Jun-N-terminal kinase (pJNK) to PDH. In other words, it may be inferred that increased phosphorylated JNK association to mitochondria may up-regulate PDK activity, thus causing increased phosphorylation (and inhibition) of PDC [49]. The mechanism on how JNK may be modulating PDK remains unclear. Other signaling pathways may also contribute to increased PDK2 expression. 

## 3 ACTIVATION AND INHIBITION OF PDKs

PDKs are activated by ATP, NADH and acetyl-CoA. They are inhibited by ADP, NAD+, CoA-SH and pyruvate [19]. PDK activation involves interaction with the E_2_ subunits of PDC to sense changes in the oxidation state and acetylation of lipoamide caused by NADH and acetyl-CoA. During starvation, PDK levels increase in most tissues, including the skeletal muscles, *via *increased gene transcription [21, 50, 51]. Under the same conditions, the amount of PDP decreases, which prevents muscles and other tissues from catabolizing glucose and gluconeogenesis precursors. The metabolism then shifts toward fat utilization [39]. Muscle protein and the supply of gluconeogenesis precursors is minimized sparing available glucose for use by the brain. Furthermore, PDC levels are emerging as important biomarkers in acute injury and neurodegeneration. In the mouse model of AD, mitochondrial bioenergetic deficits, including a decrease in PDC level and activity, were found to precede the pathological manifestation of the disease [52].

The tightly-regulated balance existing between PDK and PDP isoenzyme expression is disrupted following traumatic brain injury (TBI), which may, in turn, impair or shutdown PDC-regulated glucose metabolism. Brain PDK2 and PDP1 isoenzymes could play a critical role in the tight control of the PDHE1α1 phosphorylation/dephosphorylation cycle and activity under normal conditions. The rapid and divergent changes between PDK and PDP isoenzyme expression could render PDHE1α1 hyperphosphorylated and inactivated in the post-injury period, thus blocking PDC-regulated glucose metabolism and ATP production in TBI [36].

Glycolytic inhibitors are currently the subject of intense research in order to explore their therapeutic potential [53]. Currently, there is considerable interest in the development of specific PDK inhibitors as potential treatments for cancer, diabetes, and brain disorders. Dichloroacetate (DCA), a small-molecule inhibitor of mitochondrial PDK, activates PDH by inhibition of PDK in a dose-dependent fashion *in vitro* [54] resulting in increased delivery of pyruvate into the mitochondria. DCA down-regulates glycolysis *in vitro* and *in vivo,* and has substantial therapeutic benefit in many types of cancers [55, 56] deserving further investigation to test its therapeutic potential in brain disorders.

Regulation of PDK2 and PDK4 expression by insulin is physiologically important. PDK2 and PDK4 are both increased in conditions in which insulin levels are low (e.g., starvation and diabetes). It is observed that glucocorticoids, free fatty acids (FFAs), and insulin play important roles in setting the level of PDK expression. This level in turn determines the phosphorylation state and therefore the activation state of PDC. Thus, the inactivation of PDC that occurs in most of the major tissues of the body during starvation and diabetes is likely explained by the effects that a decrease in insulin and an increase in glucocorticoids and FFAs have on PDK4 expression under these conditions [51].

## 4 ROLE OF PDKs IN NEURON-GLIA METABOLIC COUPLING

Glial cells play a major role in brain metabolism, since they control the chemical composition of the fluid that surrounds neurons, including the levels of ions and nutrients. The structural conditions required and expressional patterns of different isomers of glucose transporter and monocarboxylate transporter have established the molecular foundation of the metabolic coupling between glia and neurons [57, 58]. The responses of the two main types of brain cells (neurons and astrocytes) to energy deprivation is a complex function of their capacity to produce ATP and the activities of various pathways involved in ion homeostasis [59]. Astrocyte processes are wrapped around synaptic contacts whereas their end-feet surround intraparenchymal capillaries and provide a cellular zone interposed between the bloodstream and other elements of the brain parenchyma. This latter structural feature has long been suggested to indicate that astrocytes participate in the transit of substances from the blood to other brain cells. The metabolisms of neurons and glial cells are interrelated, and these cells function in an integrated fashion. Several important enzymes, such as pyruvate carboxylase and glutamine synthetase, are only found in astrocytes [60, 61], and PDC plays a key role in the regulation of glucose oxidation. In order for glucose to be oxidized to CO_2_, the pyruvate formed during glycolysis must enter the TCA cycle, and this is accomplished *via *PDC in the mitochondria, as it controls the rate of pyruvate entry into the TCA cycle as acetyl coenzyme A (acetyl- CoA). PDH is inactivated by phosphorylation at its decarboxylase moiety by a tightly bound Mg^2+^/ATP-dependent protein kinase, and activated by dephosphorylation by a loosely bound Mg^2+^- and Ca^2+^-dependent phosphatase.

The control of PDHα phosphorylation is accomplished by a set of 4 different PDKs (PDK1-4) and 2 different PDPs (PDP1 and 2), which are all differentially expressed in mammalian tissues [27]. The regulation of PDC at the protein expression or activity levels contributes to the differential metabolic phenotype of neurons and astrocytes and to the directional shuttling of monocarboxylates between these cell types [62]. It has been demonstrated that all subunits of PDC are expressed in cultured astrocytes and neurons, but astrocytes express significantly higher immunoreactivities for all subunits than neurons [62]. These higher expressions of PDK2 and PDK4 in astrocytes are consistent with the higher PDHα phosphorylation status, lower PDC activity, and higher lactate production displayed by cultured astrocytes.

**Fig. (3):**
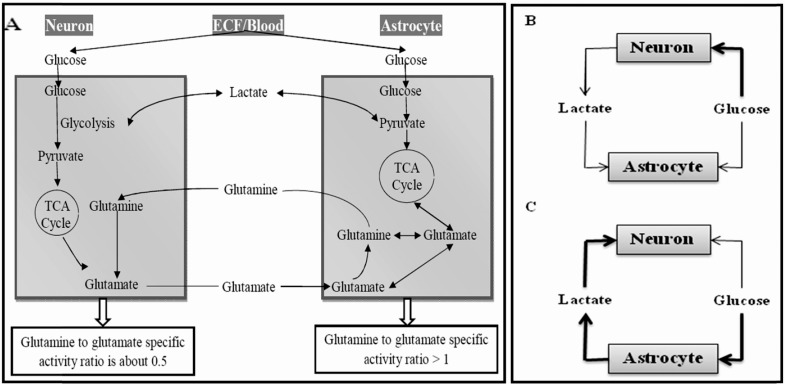
**Metabolic coupling between astrocytes and neurons**. (**A**) Neurons and astrocytes are surrounded by the ECF, which contains glucose and lactate. The glucose pool feeds the cells and is replenished by blood-derived glucose. Lactate flows from and towards the cells, and is cleared into blood at a very low rate. (**B**) The conventional model of coupling proposes the major uptake of glucose by neurons, net production of lactate by neurons, and consumption of lactate by astrocytes. (**C**) The lactate shuttle model or ANLSH proposes the major uptake of glucose by astrocytes, net production of lactate by astrocytes, and consumption of lactate by neurons (In B and C, the thickness of the arrows represents the respective fluxes). TCA, tricarboxylic acid cycle; ECF, extracellular fluid; ANLSH, astrocyte-to-neuron-lactate shuttle hypothesis.

Astrocytes are central players in neurometabolic coupling and undergo plastic adaptations in parallel with adaptive mechanisms that characterize synaptic plasticity [63-65]. The basic mechanism involves glutamate-stimulated aerobic glycolysis, that is, the sodium-coupled reuptake of glutamate by astrocytes and the ensuing activation of Na-K-ATPase, which triggers glucose uptake and processing *via *glycolysis, and results in the release of lactate from astrocytes. More often than not, neurons, astrocytes and blood vessels function in a complex network, and this metabolic linkage as well as astrocytic support are obligatory for neuronal functioning. The interstitial compartment comprises the space between endothelial cells and astrocytic end feet (the basal lamina) and also the space between neuronal and astrocytic processes. These two regions are well connected, so that no significant concentration gradients are expected between these compartments for abundant molecules like glucose and lactate [66-68]. Fig. (**3A**) schematically represents a consensual model for glucose and lactate fluxes in the mammalian neuropil. After entering neurons and astrocytes, glucose is phosphorylated by hexokinase, a reaction that in the brain is irreversible due to lack of significant glucose-6-phosphatase activity [69, 70]. There are at present two extreme views of metabolic coupling in the brain, which accommodate the higher rate of oxygen consumption and ATP synthesis in neurons, but differ sharply in the identity of the lactate source during activity, and therefore in the direction of lactate flux. The default or conventional model of neurometabolic coupling states that neurons capture most of the glucose flux and release some lactate, which is taken up by astrocytes (Fig. **3B**). This model advocates that astrocytes are more aerobic than neurons, i.e. their oxygen to glucose index (OGI) is higher. The alternative model is termed the “astrocyte-to-neuron-lactate shuttle hypothesis (ANLSH)” [71], which utters that astrocytes take most of the glucose and export it in the form of lactate, which is then taken up and oxidized by neurons (Fig. **3C**). In this model, the more aerobic cell is the neuron. Several scholarly reviews that ponder the evidence for and against the two models of neurometabolic coupling have been published in recent years [68, 72-83]. There are also mathematical models, whose conclusions obtained under different assumptions have supported one model or the other [76, 84-88]. For example, some studies imply that neurons with basal activation show no net import of pyruvate or lactate [74], while Mangia and colleagues claim just the opposite of ANLSH, that is, neurons shuttle the lactate into astrocytes, and the only way this would work in reverse (i.e. astrocyte-to-neuron) is when the astrocytic glucose transport capacity is increased 12-fold [84]. According to a recent study by Genc *et al.,* although the ANLSH is energetically more favorable for the neuron in terms of ATP produced, both under hypoxic and normoxic conditions, it is not the case of significant advantage for the astrocyte in the long term. Considering the fact that astrocytes are more resilient to hypoxia, they believe that rather than a "classical-or-ANLSH" choice for the cells, neurons and astrocytes can switch between one model or the other, depending on the energy requirements of the neuron, so as to maintain the survival of the neuron under hypoxic or glucose-and-oxygen-deprived conditions [89]. However, further investigations will surely prove useful in analyzing these models in more detail as well as in understanding such an energy demand-dependent switching [90].

## 5 ALZHEIMER'S DISEASE AND PDKs

Alzheimer’s is a neurodegenerative disease with a complex and progressive pathological phenotype characterized first by hypometabolism and impaired mitochondrial bioenergetics followed by pathological burden. Dysfunction in glucose metabolism, bioenergetics, and mitochondrial function are consistent antecedents to the development of Alzheimer pathology [52, 91-93]. A decline in brain glucose metabolism and mitochondrial function can appear decades prior to the onset of histopathological and/or clinical features and thus may serve as a biomarker of AD risk as well as a therapeutic target [94-97]. Oxidative stress and synaptic damage have been implicated in AD pathogenesis [98-103]. It is well documented that oxidative damage to mitochondrial membranes and proteins impairs mitochondrial oxidative phosphorylation efficiency and results in increased electron leak, increased H_2_O_2_ levels and higher oxidative stress [104, 105]. Key enzymes involved in mitochondrial bioenergetics, such as PDH and α-ketoglutarate dehydrogenase (αKGDH), are often the targets of oxidative modifications. This leads to decease the enzyme activity, decrease the efficiency of the mitochondrial electron transport, and increase the production of free radicals [106, 107]. In parallel with the decline in glucose metabolism in AD, there is a generalized shift away from glucose-derived energy production, which is associated with a decrease in the expression of glycolytic enzymes coupled to a decrease in the activity of the PDC [108]. Alterations in the brain metabolic profile in AD are further evidenced by concomitant activation of compensatory pathways that promote the usage of alternative substrates, such as ketone bodies, to compensate for the decline in glucose-driven ATP generation. Potentiation of mitochondrial bioenergetics and enhancement of brain glucose metabolism are expected to prevent the decline in brain glucose metabolism, promote healthy aging, and therefore prevent AD. Interestingly, PDH is an important candidate within this category whose activity can be upregulated by inhibiting the PDK. Brain lipid peroxidation and decreased brain glucose utilization are characteristics of this neurodegenerative disease [109]. Acrolein, a byproduct of lipid peroxidation that accumulates within the brain in the course of AD, decreases the PDC activity. Specifically, acrolein binds lipoic acid, a component of both PDC and αKGDH [110]. Inactivation of PDC by acrolein or other mechanisms may be at least partially responsible for mitochondrial dysfunction and impaired cerebral energy metabolism associated with AD [110-112].

## 6 BRAIN AGING AND PDKs

Whole body glucose metabolism is suppressed by the aging process. It has been reported that energy metabolism appears to decline in the brain of aging animals and this decline may be more pronounced in certain brain regions. A study focused on the presence of different level of mRNAs for PDK isoenzymes in rat brain regions has inferred that the level of PDK1 mRNA is relatively high in the cerebellum and cerebral cortex compared with the medulla oblongata and hippocampus. Aging decreases PDK1 and PDK2 mRNAs levels in the cerebellum and increases PDK2 mRNA levels in the hippocampus and cerebral cortex, whereas PDK4 mRNA expression is unaffected by aging. These results provide evidence of differences in the regional abundances of the mRNAs of PDK isoenzymes in the rat brain and that their levels are affected by aging [113].

The decrease in the neurological activities during normal brain aging has been found to be related to mitochondrial dysfunction [114]. Interest in the association between mitochondrial dysfunction and the pathobiology of aging and age-related disorders was kindled when the free radical theory of aging was posited some six decades ago. Aging and neurodegeneration are common outcomes of mitochondrial dysfunction, and are associated with a decrease in PDH activity caused by phosphorylation of its E (1α) subunit. The phosphorylation of PDH is likely to be mediated by PDK, the protein levels, and the activity, which increases with age. The age-dependent decrease and increase in ATP production and lactate accumulation, respectively, in brain tissue appear to represent a shift from aerobic glycolysis (mitochondrial PDH-dependent) to anaerobic glycolysis (cytosolic LDH-dependent). The mechanistic implications of this shift are primarily based on the inactivation of the E_1_*α* subunit of mitochondrial matrix PDH with the consequent diminished metabolism of acetyl-CoA.

Neurons in general do not survive *in vitro* in the presence of glucose, but can survive in the presence of low-molecular-weight agents, such as pyruvate, which are supplied by glial cells. It appears that neurons utilize relatively little glucose *in vivo*, and that glial cells may supply substance(s) other than glucose, for example pyruvate, as the primary source of energy [115]. Reactions catalyzed by PDC functionally link glycolysis in the cytoplasm to oxidative phosphorylation (OXPHOS) in mitochondria [116]. PDC is central to the mitochondrial fuel metabolism, and thus, to organismal health and survival. Deficiencies of PDC, due to normal aging or the impositions of congenital or acquired diseases, display strikingly similar pathologies. Therefore, PDC and its regulatory kinases, PDKs, may be potential therapeutic targets for the treatment of multiple age-related disorders of nervous system [117].

## 7 GLIOBLASTOMA AND PDKs

Malignant gliomas or malignant glial neoplasms represent the most common type of primary brain tumor and constitute a spectrum of clinicopathologic entities from low- to high-grade malignancies. Glioblastoma, having highly expressed PDK2 [118], is the most frequent and malignant of gliomas [119]. Metabolic modulation may be a viable therapeutic approach in the treatment of glioblastoma [118]. A unique metabolic signature routinely observed in cancer cells is an energy-dependence-shift from normal oxidative phosphorylation to aerobic glycolysis. In cancer cells, however, pyruvate is converted to lactate regardless of the presence of oxygen. This may be in part due to the upregulation of the PDK activity and/or inhibition of PDH in cancer cells. High rates of aerobic glycolysis can promote the malignant transformation and survival of cancer cells [120]. Recent studies have shown that an anti-apoptotic mechanism in cancer cells is mediated by aerobic glycolysis, also known as the Warburg effect. One of the major regulators of aerobic glycolysis is PDK. Enhanced glycolysis and increased lactate production is a common property of invasive cancers, which may result in the suppression of apoptosis [55, 121].

The hypoxic tumor microenvironment elevates the levels of hypoxia inducible factor (HIF). HIF is able to attenuate mitochondrial respiration through the induction of PDK1, which in part accounts for the Warburg effect that describes the propensity for cancers to avidly take up glucose and convert it to lactate with the concurrent decrease in mitochondrial respiration [122]. Similarly, normoxic overexpression of HIF1α in most, if not all, human cancers stimulates glucose uptake, glycolysis, and PDK, which inhibits PDC [123-125]. Consequently, OXPHOS is suppressed and pyruvate accumulates, which stabilizes HIF1α, creating a positive feedback loop between HIF1α and glycolysis [120, 126], which can be reversed by the knockdown of PDK [127]. DCA inhibition of PDK frees up the mitochondrial gate-keeping enzyme PDH which is then able to convert pyruvate to acetyl-CoA and initiate normal oxidative phosphorylation *via *the Krebs cycle [55]. These findings suggest that the PDK/PDH axis may contribute to glioma cell metabolism and tumor growth.

## 8 BRAIN INJURY AND PDKs

While oxidative damage to cerebral energy metabolism is a critical contributor to delayed and necrotic neuronal death, oxidative stress is also a powerful initiator of apoptosis, which also contributes significantly to ischemic neural cell death. Mitochondria normally generate reactive oxygen species and contribute significantly to the elevated net production of these destructive agents during reperfusion. The mitochondria are both targets and sources of oxidative stress. This dual relationship is particularly evident in experimental paradigms modeling ischemic brain injury. Mitochondria play central roles in acute brain injury. Following TBI, the degree of mitochondrial injury or dysfunction can be an important determinant of cell survival or death [128]. Alterations in mitochondrial respiratory capacity have been demonstrated following TBI in adult animals and humans [129-131]. Dysregulated glucose metabolism and energy failure is a metabolic characteristic and an indicator of poor prognosis for patients with severe TBI [129, 132-137]. Recent studies suggest an important role for PDH in the altered brain energy metabolism following TBI [138-141]. Brain PDK and PDP isoenzymes appear to be very sensitive to the effects of traumatic injury: controlled cortical impact-induced TBI (CCI-TBI) and craniotomy induced a marked increase in PDK protein expression and a reduction in PDP protein expression, which would favor increased PDHE1α1 phosphorylation and inhibition with subsequent uncoupling of PDH-regulated glucose metabolism. Furthermore, all of the four PDK isoenzymes appear to be involved in altered PDHE1α1 phosphorylation and activity at different stages after TBI due to their distinct spatiotemporal induction patterns. Divergent changes in PDK and PDP isoenzyme expression were consistently observed at various times following CCI-TBI [36]. How the post-injury cortical spreading depression (CSD) is, however, correlated with PDK/PDP expression in TBI remains unanswered.

## 9 CONCLUSIONS AND FUTURE PERSPECTIVES

Efficient cerebral activity requires an adequate energy supply for each of the cellular mechanisms involved. The concept of metabolic coupling between neurons and glia, particularly astrocytes, in the context of maintaining energy metabolism homeostasis in the brain has been thought about for some time. Furthermore, astrocytic and neuronal metabolisms are closely coupled to fulfill the anabolic and energy needs associated with brain activation. Glial cells play a major role in brain metabolism, by controlling the chemical composition of the fluid that surrounds neurons. Structural relationships between astrocytes and other elements of nervous tissue are of particular relevance in this discussion of brain energy metabolism at the cellular level. Enhanced understanding of neuron–astrocyte metabolic interactions offers a potential means for developing novel therapeutic strategies for many neurological disorders associated with metabolic deficits. The dedicated PDK/PDP system responds to metabolite and hormone signals that vary the PDC activity in response to changes in nutritional states. PDKs are involved in the regulation of glucose oxidation, which could ultimately affect whole body glucose metabolism. The metabolisms of neurons and glial cells are interlinked, and these cells function in an integrated fashion. Furthermore, the regulation of PDC protein expression or activity contributes to the metabolic phenotypes of neurons and astrocytes and to the directional shuttling of monocarboxylates between these cells. Up to now, the astrocyte–neuron lactate shuttle hypothesis still promises to help unravel important cellular and molecular aspects of neurometabolic coupling. Astrocytes are central players in this coupling as they take up glucose from blood vessels for neurons. On the other hand, PDC activity is tightly regulated by the phosphorylation by four isoforms of PDKs. There is mounting evidence of crosstalk between astroglial and neuronal cells in terms of maintaining the energy requirements for neurological activities. 

Metabolic disorders based on the PDH/PDK system cause defects in the synthesis, metabolism, transportation, and storage of biochemical compounds, which in turn may lead to tissue intoxication or energy deficiency in the brain resulting in life threatening neuro-metabolic disorders in addition to dysfunction of several other vital organs. The precise role played by PDKs in the nervous system, especially in glial-neuron interactions remains unclear, and much remains to be determined regarding how PDKs participate in signaling pathways and the metabolic control of glia-neuron interactions. Further investigations are required to verify the role played by PDKs in the nervous system, especially in glia-neuron interactions, and to investigate the behaviors of PDKs and their corresponding microRNAs. We are convinced that such studies will lead to a major breakthrough in neuroscience research and establish new dimensions for translational research into disorders of the nervous system.
